# Latent Class Analysis Identifies Four Distinct Patient Deprescribing Typologies Among Older Adults in Four Countries

**DOI:** 10.1093/geroni/igaf002

**Published:** 2025-01-17

**Authors:** Kristie Rebecca Weir, Vincent D Marshall, Sarah E Vordenberg

**Affiliations:** Sydney School of Public Health, Faculty of Medicine and Health, University of Sydney, Sydney, NSW, Australia; Institute of Primary Health Care (BIHAM), University of Bern, Bern, Switzerland; University of Michigan College of Pharmacy, Ann Arbor, Michigan, USA; University of Michigan College of Pharmacy, Ann Arbor, Michigan, USA

**Keywords:** Attitudes, Deprescription, Health care communication, Medicine, Patient preferences

## Abstract

**Background and Objectives:**

Polypharmacy, the concurrent use of multiple medicines, is a growing concern among older adults and those with chronic conditions. Deprescribing through dose reduction or discontinuing selected medicines is a strategy for reducing medicine-related harm. The Patient Deprescribing Typology was developed using qualitative methods to describe the varying factors that are important to older adults when they consider deprescribing. The objective of this study was to use quantitative methods to define distinct classes of older adults via the Patient Deprescribing Typology.

**Research Design and Methods:**

This study used a cross-sectional experimental design in which data was collected via an online survey from participants 65 years and older in Australia, the Netherlands, the United Kingdom, and the United States. A latent class analysis was performed using the 4-item Patient Deprescribing Typology that collected data about the beliefs about the importance of medicines, how older adults learn about medicines, medicine decision-making preferences, and attitudes towards stopping medicines.

**Results:**

Older adults (*n* = 2,250) were a median of 70 years and 2-thirds reported that their highest level of education was an associate’s degree or trade school or less. We identified 4 distinct Patient Deprescribing Typology classes: Class 1 “Trusts their doctor” (41.6%), Class 2 “Makes own decisions” (30.2%), Class 3 “Avoids deprescribing” (15.5%), and Class 4 ‘Medicines not important’ (12.7%).

**Discussion and Implications:**

Older adults report diverse perspectives about deprescribing, emphasizing the need for tailored communication strategies in clinical settings. Additional research is needed to examine older adults’ preferences in real-world contexts to refine and improve deprescribing interventions.

**Clinical Trial Registration:**

NCT04676282

Translational Significance: This study used the Patient Deprescribing Typology to identify distinct groups of older adults based on their beliefs and preferences regarding medication use and deprescribing. Through an online survey of adults aged 65+ from several countries, four classes emerged: those who trust their doctor, make their own decisions, avoid deprescribing, or find medicines unimportant. These insights emphasize the need for tailored communication strategies in clinical settings to better support patient-centered deprescribing.

## Background and objectives

Polypharmacy, the concurrent use of multiple medicines, is a growing concern among older adults and those with chronic conditions (Kantor et al., 2015). As the number of prescribed medicines increases, so does the risk of adverse drug reactions, drug interactions, and medicine nonadherence (Fried et al., 2014). Deprescribing through dose reduction or discontinuing selected medicines is a strategy for reducing medicine-related harm (Scott et al., 2015). However, implementing deprescribing decisions in clinical practice is challenging.

Research has shown that a significant proportion of patients decline opportunities to reduce their medicines. In fact, up to 75% of older adults choose not to participate in deprescribing intervention studies (Kutner et al., 2015; McCarthy et al., 2023). Two recent European cluster-randomized trials highlighted significant challenges in deprescribing uptake. In a trial conducted in Switzerland (Zechmann et al., 2020), approximately one-quarter of patients (22 out of 87) declined their general practitioner’s (GP’s) offer to deprescribe a medicine, even when a shared decision-making intervention was used. Similarly, a study in the Netherlands (Luymes et al., 2018) found that 35% of participants in the intervention group did not attempt to deprescribe their medicine. Possible reasons for this include patients’ positive attitudes toward their medicines, believing them to be beneficial and necessary, as well as fears about the potential consequences of deprescribing (Weir et al., 2023).

Effective tailored communication between health care providers and patients is essential for successful deprescribing and fostering collaborative decision-making (Jansen et al., 2016). Individual attitudes toward medicines vary, which may influence responses to deprescribing recommendations (Gillespie et al., 2018). To understand these differences, researchers have developed typologies categorizing patients based on their medicine-related beliefs and behaviors. Previous studies of typologies have primarily focused on specific medicine classes, decision-making, or general medicine management (Flynn et al., 2006; Clare H Luymes et al., 2017; Møller et al., 2021; Wrede-Sach et al., 2013). Earlier qualitative work revealed complex and often contradictory attitudes among older adults, leading to the development of a Patient Deprescribing Typology (Weir et al., 2018) considering medicine beliefs, deprescribing preferences, and decision-making styles. Although this typology has been applied in various studies (Crutzen et al., 2020; Goyal, 2023; Lüthold et al., 2022), quantitative methods are needed for broader implementation. Previous quantitative research explored deprescribing typologies in a static way (Weir et al., 2023); however, this study is the first to use latent class analysis (LCA) to identify distinct patient groups within the data. The objective of this study was to define distinct latent classes of older adults via the Patient Deprescribing Typology—this study was part of a larger survey-based online experiment. For clarity, we use “Typology” to refer to the previous qualitative and quantitative work that led to the formation of the Patient Deprescribing Typology and “Class” to refer to the current work associated with the LCA.

## Research Design and Methods

### Development of the Patient Deprescribing Typology

This section provides an overview of the qualitative and quantitative studies that form the evidence base for the Patient Deprescribing Typology (Table 1). Stages 1, 2, and 3 present a concise summary of the authors’ previously published work that underpins the development of the typology.

**Table 1.** Development of the Patient Deprescribing Typology

	Qualitative exploration (Weir et al., 2018)	Quantitative exploration (Weir et al., 2024a)
Objective	To explore the reasons behind variation in older adult attitudes and preferences related to medicine deprescribing.	To develop a quantitative Patient Deprescribing Typology measure that could be incorporated into research and practice.
Study population	Adults 75 years and older (*n* = 30) and companions (*n* = 15) in Australia.	Adults 65 years and older (*n* = 4,688) in Australia, the Netherlands, the United Kingdom, and the United States.
Methodology	In-depth semi-structured interviews followed by a framework analysis, guided by a theoretical shared decision-making framework (Jansen et al., 2016)	An online survey asked participants to select one of the three overarching Patient Deprescribing Typologies. The primary outcome was summarized using descriptive statistics.
Results	Three typologies were identified: “Attached to medicines”: Positive attitudes towards medicines and prefer to defer medicine-related decisions to their doctor. “Would consider deprescribing”: Ambivalent attitudes towards medicine but preferred a proactive role in decision-making while being open to deprescribing. “Defers to others”: Lacked knowledge about medicines and deferred decisions to their doctor or companion.	Participants selected one overarching typologies that described them best: “Attached to medicines”: *n* = 1,446 (30%) “Would consider deprescribing”: *n* = 2,464 (53%) “Defers (medicine decision-making) to others”: *n* = 778 (17%) The quantitative results were generally consistent with our qualitative work, with few discrepancies.
Strengths	A heterogeneous sample of community-dwelling older adults and companions varying in age, education, levels of comorbidities, health status, and polypharmacy.	A large sample of older adults across four countries with different health care systems.
Limitations	Sample number of participants within one country.	Underrepresentation of older adults with poor health status. Participants may not have agreed with all the statements in the selected typology.

#### Stage 1: qualitative exploration

Weir et al. conducted qualitative work to explore the nuances of deprescribing and decision-making preferences with older adults (*n* = 30) and their caregivers (*n* = 15) from Australia (Weir et al., 2018). Participants varied considerably across key themes such as attitudes toward medicines and deprescribing, patient–doctor trust, knowledge, and decision-making preferences, leading to the identification of three participant typologies. As part of the process, participants responded to validated measures including decision-making preferences (Control Preferences scale; Degner et al., 1997), self-assessed quality of life and general health (DeSalvo et al., 2006), comorbidities (Charlson Comorbidity Index; Charlson et al., 1987), and activities of daily living (Katz, 1983). This qualitative work involved participants with varying levels of frailty and health status reflecting on their medicines. Of note, the typologies were not always mutually exclusive, as some participants displayed traits overlapping between groups. Within each category, attitudes varied slightly depending on individual concerns about specific medicines or health conditions.

- ***Typology 1*** “Attached to medicines” had positive attitudes toward medicines, high trust in their doctor, they left decisions to their doctor, and were resistant to deprescribing. They had some knowledge about their health or medicines and reported good self-rated health.
- ***Typology 2*** “*Would consider deprescribing”* held ambivalent attitudes towards their medicines, preferred a proactive role in decision-making, and were open to deprescribing. They were generally knowledgeable about their health or medicines and accessed information. They reported very good or higher self-rated health.
- ***Typology 3*** “Deferred decision-making to others” gave medicines little thought and deferred decisions to their doctor or companion, they were generally unaware that deprescribing was an option. They perceived they lacked knowledge about their health or medicines. The majority were male, frailer and reported fair or poor self-rated health.

#### Stage 2: developing the measure

Next, we developed a quantitative Patient Deprescribing Typology typology measure through an iterative process involving experts and patient feedback (Weir, 2020). The measure incorporated key aspects of the three qualitatively derived Patient Typology categories. Input was gathered from 10 multidisciplinary experts, including specialists in geriatrics, general practice, pharmacy, health literacy, ethics, health psychology, shared decision-making, and a consumer representative. The measure was then pilot-tested in Australia with seven older adults and two caregivers, leading to minor wording adjustments for clarity (Table 2).

**Table 2.** Medicine Decision-Making Preferences by Domain

Domain	Statement	Keywords
Beliefs about importance	My medicines are doing what they are supposed to do.	Effective
	My medicines are important. They keep me alive and help me to live well.	Important
	I don’t really care much about my medicines. I take them as my doctors tells me to.	Not important
Learning style	I know about my medicines. I ask my doctor or read the information leaflet or search online.	Multiple sources
	My doctor and I talk about my medicines together.	Discuss with doctor
	I don’t know much about my medicines.	Not informed
Decision-making preferences	I trust my doctor to make decisions about my medicines.	Decision by doctor
	I make decisions about the medicines I take or share the decision with my doctor.	Decision by self
	Other people (e.g., my doctor or companion) make decisions about medicines for me.	Decision by other
Attitudes towards deprescribing	If my doctor said I could stop a medicine, I think that would be ok.	Open to deprescribing
	I wish I did not have to take as many medicines, and would stop taking one or more if I could.	Prefer deprescribing
	I would not want to stop taking any of my medicines.	Continue medicines

#### Stage 3: quantitative exploration

The quantitative Patient Deprescribing Typology measure was incorporated into a hypothetical vignette study conducted across four countries (AU, NL, UK, US; Weir et al., 2024a). In total, 4,688 participants (88% of the final sample) completed the patient typology question. However, participants could only select one of three predetermined typology descriptions, which may have limited the depth of the analysis. The survey focused on two medicine types: proton pump inhibitor and simvastatin and approximately half of participants had experience with one of these medicine types. This study validated the quantitative Patient Typology measure by examining associations between patient characteristics and typology categories. The quantitative findings generally aligned with the qualitative hypothesis although there were some discrepancies between the qualitative and quantitative results. Approximately one-half of participants selected Typology 2 “Would consider deprescribing” (53%), followed by Typology 1 “Attached to medicines” (31%), and Typology 3 “Defers decision-making to others” (17%). A limitation of this study is that trust in the doctor was not included as a separate measure for decision-making preferences.

#### Latent class analysis

This current study was part of a larger survey-based online experiment testing contextual factors influencing older adults’ preferences for deprescribing simvastatin (Weir et al., 2024b). Participants were included if they were 65 years and older and lived in Australia (AU), the Netherlands (NL), the United Kingdom (UK), and the United States (US) (Weir et al., 2024b). Qualtrics Research Panels (Provo, UT) recruited participants from August 9 to September 20, 2021. We aimed for a sample size of 1,200 participants per country, based on previous research (Vordenberg et al., 2022), with sampling quotas employed to ensure roughly equal representation of each country and by gender. Quotas were established for race and ethnicity in the United States to ensure the data was representative of national census data. The survey was administered in English (AU, UK, and US) and Dutch (NL).

Participants initially read a vignette about a 76-year-old patient taking 11 medicines to manage multiple health conditions. During a routine medical appointment, the primary care provider (PCP) recommended stopping simvastatin (control) (Vordenberg et al., 2022). Participants were randomized to one of six contextual factors that may affect agreement with stopping the medicine: (1) control, PCP recommends stopping simvastatin (2) a cardiologist initially prescribed the medicine, (3) her adult daughter believes simvastatin is important, (4) her spouse had experienced a stroke after stopping simvastatin, (5) there was an educational poster about strokes in the waiting room, or (6) she acknowledged that it would be challenging to engage in the lifestyle changes that would be beneficial if the simvastatin were to be stopped. Randomization was built into the Qualtrics survey so that each scenario was evenly presented to participants. Participants only received one vignette and therefore were unaware of the randomization. However, due to a programming error, only participants who made decisions for the hypothetical patient were included in this study. The variables we measured included:

- *Patient Deprescribing Typology*: We sought to determine whether there were additional typologies within the Patient Deprescribing Typology. Therefore, we asked participants four medicine-related questions that were derived from qualitative and quantitative exploration of the Patient Deprescribing Typology. For each question, participants selected one response that best aligned with their attitudes, beliefs, and preferences. Keywords were identified to simplify the reporting of the results (Table 2). We also asked participants questions based on hypothesized relationships with the qualitatively developed Patient Deprescribing Typology (Weir et al., 2018) and subsequent quantitative exploration (Weir et al., 2024a), a systematic review and meta-analysis of individuals’ attitudes toward deprescribing (Weir et al., 2022), and previous deprescribing research conducted on communication and shared decision making (Ailabouni et al., 2022; Fajardo et al., 2019; Jansen et al., 2016).
- *Agreement with stopping simvastatin*: The participants’ attitude toward stopping simvastatin as measured by the extent of agreement with the statement, “I think that Mrs. EF should follow the doctor’s recommendation and stop taking simvastatin,” on a 6-point Likert scale with “strongly disagree” (1) and “strongly agree” (6) as the scale anchors.
- *Risk perceptions of stopping simvastatin*: We adapted the 6-item Tripartite Model of Risk Perception (TRIRISK) scale that measures Deliberative, Affective, and Experiential risk perceptions with questions such as, “How likely do you think it is that Mrs. EF’s heart health will worsen at some point in the future without simvastatin?” with “very unlikely” (1) and “very likely” (7) as the scale anchors (Ferrer et al., 2016).
- *Attitude toward stopping simvastatin*: We adapted a single-item measure, “I think that Mrs. EF stopping simvastatin would be…” with “very negative” (1) and “very positive” (10) as the scale anchors (Dormandy et al., 2006; Scherer et al., 2018).
- *Trust in primary care doctor*: The 10-item Wake Forest Physician Trust Scale measured individuals’ trust in their primary care provider on a 5-point scale, with “strongly disagree” (1) and “strongly agree” (5) as the scale anchors (Hall et al., 2002).

Finally, we also collected information about age, gender, country of residence, highest level of education, and current or previous personal experience with taking a HMG co-A reductase inhibitor (“statin”).

This study was registered as a clinical trial at Clinicaltrials.gov Identifier: NCT04676282. This study received exempt status approval from the University of Michigan Health Sciences and Behavioral Sciences Institutional Review Board (HUM00183129).

#### Statistical analysis

We conducted a LCA to identify subgroups of older adults related to the Patient Deprescribing Typology (Hagenaars & McCutcheon, 2002). Our methodology included data summaries using mean and standard deviation (*SD*) or median and interquartile range (IQR) for numeric variables, depending on normality, and for categorical variables, we used counts and percentages. Our initial analysis looked for latent classes within our typology variables, adjusted by a multiple regression model. We determined the number of potential latent classes by testing different numbers and then comparing them with the Bayesian Information Criterion (BIC), a common model fit based on the log-likelihood of the models, adjusted for the number of covariates. We used the minimum BIC as the deciding factor. We also ran 100 iterations for each possible number of latent classes to avoid selecting local maxima of the model likelihood, instead of a global maximum, a common problem with latent class estimation models. Our model adjustment for the search for the number of latent classes and the model adjustment for the final classification of our respondents was a one-step model, where the covariates were employed in determining the classes rather than in a separate model afterward. Once we had determined the latent class distribution, we reexamined our data with descriptive statistics. We also conducted a linear regression on deprescribing attitudes to determine how the latent classes and contextual factors predicted that outcome. In a table, we included the multinomial logit model used in the LCA classification of the population. Statistical analyses were performed with R statistical software (v. 4.2.2; Team, 2020). The poLCA package in R was used in the estimation of latent classes (Linzer & Lewis, 2011).

### Results

A total of 5,400 panelists clicked on the survey link. Participants were excluded if they resided outside the four target countries (*n* = 170), did not report their age (*n* = 5), did not agree to provide high-quality responses (*n* = 5), did not complete the survey (*n* = 346), or completed the survey in less than 5 min (median completion time = 14.5 min), giving a completion rate of 93.2% among eligible participants. For this study, we excluded participants who were asked to make a hypothetical decision for themselves (*n* = 2,477) due to a programming error resulting in incomplete data collection for this sample. We excluded participants (*n* = 222) who did not answer all the survey questions described earlier as missing data was dropped when conducting a LCA. We excluded individuals who identified as a gender other than male or female as the small sample size (*n* = 5) prevented us from placing them in a latent class. Our final analytical sample included 2,250 participants from Australia, the Netherlands, the United Kingdom, and the United States.

#### Overall

The median age of the participants was 70 years, and two-thirds reported that their highest level of education was an associate’s degree or trade school or less (Table 3). More than half of the participants had personal experience taking statins, either currently (47.2%) or in the past (8.8%).

**Table 3.** Demographic Characteristics and Medicine-Related Attitudes of Survey Participants (*n* = 2,250)

Variable	*N* (%)	Mean (*SD*)
*Demographic characteristics*		
Age in years, median (interquartile range)	70 (67, 74)	
Gender		
Male	1,130 (50.2)	
Female	1,120 (49.8)	
Country		
Australia	570 (25.3)	
Netherlands	532 (23.6)	
United Kingdom	588 (26.1)	
United States	560 (24.9)	
Education level		
High school diploma or less	718 (31.9)	
Trade school, some college, or associate’s degree	744 (33.1)	
Bachelor’s degree	516 (22.9)	
Master’s degree or higher	272 (12.1)	
Personal experience with HMG-CoA reductase inhibitor (statin)		
Current	1,062 (47.2)	
Previous	197 (8.8)	
Never	991 (44.0)	
*Medicine decision making preferences*		
Beliefs about importance		
Effective	1,355 (60.2)	
Important	474 (21.1)	
Not important	421 (18.7)	
Learning style		
Multiple sources	1,054 (46.8)	
Discuss with doctor	1,051 (46.7)	
Not informed	145 (6.4)	
Decision making preferences		
Decision by doctor	1,289 (57.3)	
Decision by self	94 (41.9)	
Decision by others	19 (0.8)	
Attitudes towards stopping		
Open to deprescribing	1,260 (56.0)	
Prefer deprescribing	663 (29.5)	
Continue medicines	327 (14.5)	
*Vignette-based experiment*		
Agreement with stopping medicine by contextual factors ^a^		
Control (*n* = 375)		4.8 (1.3)
Specialist prescriber (*n* = 388)		4.3 (1.5)
Family influence (*n* = 368)		4.7 (1.3)
Spouse stroke (*n* = 380)		4.1 (1.5)
Stroke image (*n* = 368)		4.5 (1.4)
Lifestyle change difficult (*n* = 371)		4.7 (1.3)
Attitudes and risk perceptions		
Attitude towards stopping medicine ^b^		6.6 (2.3)
Anxious or worried that health will worsen without medicine ^c^		4.4 (2.2)
Trust in doctor ^d^		3.5 (0.4)

^a^1 = strongly disagree, 6 = strongly agree.

^b^1 = very negative, 10 = very positive.

^c^1 = very unlikely, 7 = very likely.

^d^1 = strongly disagree, 5 = strongly agree.

When asked about the importance of medicines, participants most frequently referred to the medicine’s effectiveness (60.2%). Participants were evenly divided between using multiple sources of information (46.8%) and discussing with their doctor (46.7%) to learn about their medicines. Most participants deferred to their doctor regarding medicine-related decisions (57.3%). Participants were generally open to the idea of deprescribing (56.0%) or preferred to deprescribe medicines if possible (29.5%). Participants reported moderate levels of trust in their doctor as well as negativity, anxiety, or worry that their health will worsen in the future without simvastatin.

#### Demographic characteristics of latent classes

**Figure 1.**
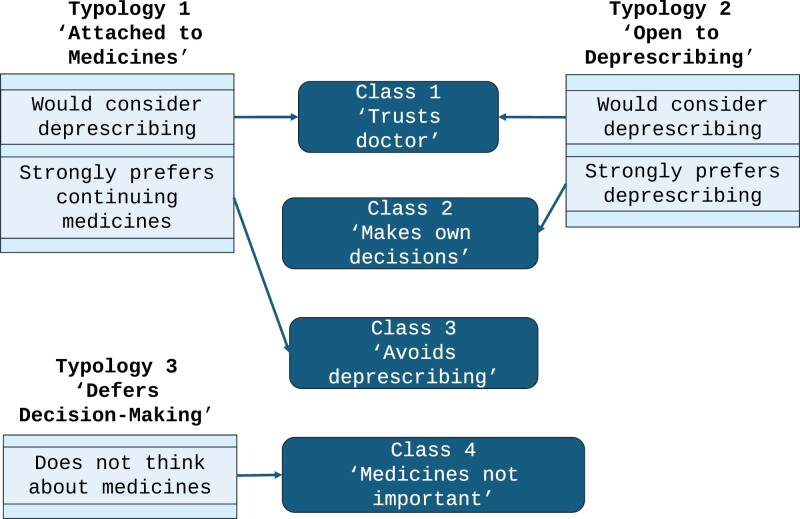
Probability of latent class membership by level of agreement with deprescribing in hypothetical vignette.

We identified four latent classes in the patient deprescribing typology (Table 4, Figure 1). Participants most frequently reported trusting their doctor and being open to stopping medicines (Class 1 “Trusts their doctor,” *n* = 935, 41.6%), followed by making their own decisions about medicines (Class 2 “Makes own decisions,” *n* = 680, 30.2%), preferring not to stop any of their medicines (Class 3 “Avoids deprescribing,” *n* = 349, 15.5%), and believing their medicines were not important (Class 4 “Medicines not important,” *n* = 286, 12.7%).

**Table 4.** Demographic Characteristics and Medicine-Related Attitudes of Survey Participants by Latent Class (*n* = 2,250)

Variable	Class 1: trusts their doctor (*n* = 935, 41.6%)	Class 2: makes own decisions (*n* = 680, 30.2%)	Class 3: avoids deprescribing (*n* = 349, 15.5%)	Class 4: medicines not important (*n* = 286, 12.7%)	*p*-Value
*Demographic characteristics*					
Age in years, median (interquartile age)	71 (68–76)	70 (67–73)	70 (67–74)	70 (67–74)	**<.001**
Gender, *N* (%)					**<.001**
Male	578 (61.8)	235 (34.6)	143 (41.0)	174 (60.8)	
Female	357 (38.2)	445 (65.4)	206 (59.0)	112 (39.2)	
Country, *N* (%)					
Australia	333 (35.6)	122 (17.9)	79 (22.6)	36 (12.6)	**<.001**
Netherlands	106 (11.3)	235 (34.6)	61 (17.5)	130 (45.5)	
United Kingdom	202 (21.6)	148 (21.8)	142 (40.7)	96 (33.6)	
United States	294 (31.4)	175 (25.7)	67 (19.2)	24 (8.4)	
Education level, *N* (%)					**<.001**
Less than a Bachelor’s degree	596 (63.7)	375 (55.1)	251 (71.9)	240 (83.9)	
Bachelor’s degree or higher	339 (36.3)	305 (44.9)	98 (28.1)	46 (16.1)	
Personal use of statin, *N* (%)					
Current or previous	576 (61.6)	336 (49.4)	225 (64.5)	122 (42.7)	**<.001**
Never	359 (38.4)	344 (50.6)	124 (35.5)	164 (57.3)	
*Medicine decision making preferences*					
Beliefs about importance, *N* (%)					
Effective	565 (60.4)	494 (72.6)	194 (55.6)	102 (35.7)	**<.001**
Important	272 (29.1)	59 (8.7)	143 (41.0)	0 (0)	
Not important	98 (10.5)	127 (18.7)	12 (3.4)	184 (64.3)	
Learn style, *N* (%)					
Multiple sources	212 (22.7)	533 (78.4)	177 (50.7)	132 (46.2)	**<.001**
Discuss with doctor	716 (76.6)	146 (21.5)	155 (44.4)	34 (11.9)	
Not informed	7 (0.7)	1 (0.1)	17 (4.9)	120 (42.0)	
Decision-making preferences, *N* (%)					
Decision by doctor	771 (82.5)	57 (8.4)	226 (64.8)	235 (82.2)	**<.001**
Decision by self	163 (17.4)	617 (90.7)	123 (35.2)	39 (13.6)	
Decision by others	1 (0.1)	6 (0.9)	0 (0)	12 (4.2)	
Attitudes toward stopping, *N* (%)					
Open to deprescribing	720 (77.0)	303 (44.6)	3 (0.9)	234 (81.8)	**<.001**
Prefer deprescribing	142 (15.2)	357 (52.5)	113 (32.4)	51 (17.8)	
Continue medicines	73 (7.8)	20 (2.9)	233 (66.8)	1 (0.3)	
*Vignette-based experiment*					
Agreement with stopping medicine by contextual factors,^a^ *N* (%)					
Overall	4.93 (1.16)	4.57 (1.30)	3.09 (1.49)	4.60 (1.24)	**<.001**
Control	5.13 (1.09)	4.89 (1.14)	3.12 (1.35)	4.92 (1.13	**<.001**
Specialist prescriber	4.78 (1.29)	4.23 (1.44)	2.95 (1.43)	4.46 (1.31)	
Family influence	4.94 (1.19)	4.79 (1.00)	3.08 (1.61)	5.08 (0.83)	
Spouse stroke	4.63 (1.17)	4.14 (1.49)	2.84 (1.46)	4.44 (1.42)	
Stroke image	4.96 (1.19)	4.42 (1.39)	3.43 (1.62)	4.27 (1.25)	
Lifestyle change difficult	5.13 (0.97)	4.96 (1.01)	3.26 (1.43)	4.54 (1.20)	
Attitudes and risk perceptions, *N* (%)					
Attitude toward stopping medicine (*SD*) ^b^	7.34 (2.04)	6.79 (2.00)	3.94 (2.05)	6.76 (1.73)	**<.001**
Anxious or worried that health will worsen without medicine ^c^	3.81 (1.96)	4.05 (1.97)	6.94 (1.82)	3.88 (1.86)	**<.001**
Trust in doctor ^d^	3.66 (0.32)	3.27 (0.44)	3.51 (0.39)	3.33 (0.44)	**<.001**

*Note*: Statistically significant at *p* < .05 and are reported in bold font.

^a^1 = strongly disagree, 6 = strongly agree.

^b^1 = very negative, 10 = very positive.

^c^1 = very unlikely, 7 = very likely.

^d^1 = strongly disagree, 5 = strongly agree.

“Trusts their doctor” (Class 1) participants were most often male (61.8%), from Australia (35.6%), and currently or previously took a statin (61.6%). Over one-third of participants (36.3%) reported earning a Bachelor’s degree or higher. Participants thought that their medicines were effective (60.4%), they discussed their medicines with their doctor (76.6%), they trusted their doctor to make decisions about their medicines (82.5%), and they were open to deprescribing (77.0%).

“Makes own decisions” (Class 2) participants were most often female (65.4%), from the Netherlands (34.6%), and one-half (49.4%) reported currently or previously taking a statin. “Makes own decisions” (Class 2) participants most frequently reported earning a Bachelor’s degree or higher (44.9%). Participants reported that their medicines were effective (72.6%), they used multiple sources of information to learn about medicines (78.4%), they made decisions about their medicines (90.7%), and they preferred to deprescribe medicines if possible (52.5%).

Participants in Class 3: “Avoids deprescribing” were most frequently female (59.0%), from the United Kingdom (40.7%), and nearly two-thirds (64.5%) reported current or previous experience with statins. About one-quarter of participants (28.1%) reported earning a Bachelor’s degree or higher. Participants reported that medicines were effective (55.6%), they used multiple sources to learn about medicines (50.7%), and they trusted their doctor to make medicine-related decisions (64.8%). However, most “Avoids deprescribing” (Class 3) participants preferred to continue their medicines (66.8%).

Class 4 “Medicines not important” participants were most often male (60.8%), from the Netherlands (45.5%), and had less experience currently or previously taking statins (42.7%). Participants (16.1%) infrequently reported earning a Bachelor’s degree or higher. Participants reported that their medicines were not important (64.3%), they used multiple sources to learn about medicines (46.2%), they deferred to their doctor for medicine-related decisions (82.2%), and they were open to deprescribing (81.8%).

#### Comparison of attitudes and risk perceptions by latent class

We compared each latent class to Class 1 “Trusts their doctor” as it included the largest number of participants (Table 5). Compared with participants in Class 1 “Trusts their doctor,” participants in Class 2 “Makes own decisions” reported less positive attitudes toward stopping medicines (OR 0.88, 95% CI .79, .98) and less trust in their doctor (OR 0.07, 95% CI .04, .12). Participants in Class 3 “Avoids deprescribing” reported less positive attitudes towards stopping medicines (OR 0.65, 95% CI 0.57, 0.74), more anxiety or worry that their health will worsen in the future without simvastatin (OR 1.49, 95% CI 1.28, 1.74), and decreased trust in their doctor (OR 0.34, 95% CI .18, .67). Participants in Class 4 “Medicines not important” were less likely to be anxious or worried that their health would worsen in the future without the simvastatin (OR 0.85, 95% CI 0.74, 0.96) and reported less trust in their doctor (OR 0.08, 95% CI 0.05, 0.15).

**Table 5.** Multinomial Logit Model Comparing Deprescribing Latent Classes

Variable	Class 2 “Makes own decisions” vs Class 1 “Trusts their doctor”		Class 3 “Avoids deprescribing” vs Class 1 “Trusts their doctor”		Class 4 “Medicines not important” vs Class 1 “Trusts their doctor”	
	OR (95% CI)	*p*-Value	OR (95% CI)	*p*-Value	OR (95% CI)	*p*-Value
*Demographic characteristics*						
Age in years	0.93 (0.89, 0.96)	**<.001**	0.99 (0.95, 1.04)	.721	0.97 (0.92, 1.01)	.138
Gender						
Male	Ref	Ref	Ref	Ref	Ref	Ref
Female	3.50 (2.36, 5.17)	**<.001**	2.41 (1.44, 4.04)	**.001**	1.20 (0.74, 1.93)	.458
Country						
Australia	0.73 (0.44, 1.19)	.200	0.81 (0.39, 1.67)	.566	1.20 (0.56, 2.56)	.634
Netherlands	5.71 (3.29, 9.93)	**<.001**	2.90 (1.27, 6.61)	**.012**	12.51 (5.80, 26.95)	**<.001**
United Kingdom	1.24 (0.74, 2.07)	.407	3.08 (1.52, 6.22)	**.002**	4.13 (1.99, 8.58)	**<.001**
United States	Ref	Ref	Ref	Ref	Ref	Ref
Education level						
Less than a Bachelor’s degree	0.62 (0.42, 0.91)	**.013**	1.02 (0.60, 1.75)	.940	2.66 (1.50, 4.71)	**.001**
Bachelor’s degree or higher	Ref	Ref	Ref	Ref	Ref	Ref
*Attitudes and risk perceptions*						
Attitude toward stopping medicine ^a^	0.88 (0.79, 0.98)	**.020**	0.65 (0.57, 0.74)	**<.001**	0.88 (0.77, 1.00)	.052
Anxious or worried that health will worsen without medicine ^b^	0.92 (0.83, 1.03)	.173	1.49 (1.28, 1.74)	**<.001**	0.85 (0.74, 0.96)	**.011**
Trust in doctor ^c^	0.07 (0.04, 0.12)	**<.001**	0.34 (0.18, 0.67)	**.002**	0.08 (0.05, 0.15)	**<.001**

*Notes*: CI = confidence interval; OR = odds ratio. Statistically significant at *p* < .05 and are reported in bold font.

^a^1 = very negative, 10 = very positive.

^b^1 = very unlikely, 7 = very likely.

^c^1 = strongly disagree, 5 = strongly agree.

#### Hypothetical vignette

**Figure 2.**
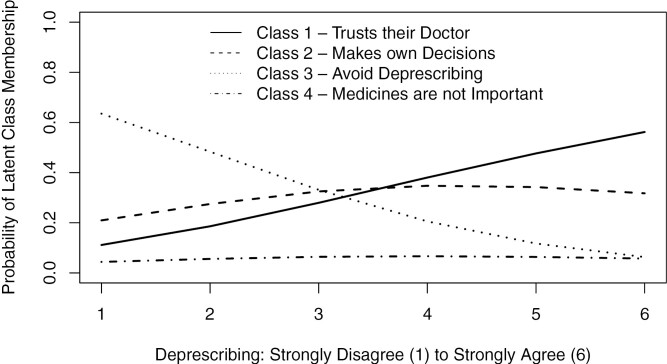
Relationship between the latent classes and typologies from the previous qualitative and quantitative research.

Participants were asked to rate their level of agreement with stopping simvastatin as part of a hypothetical vignette resulting in mean scores (*SD*) ranging from 3.09 (1.49) for Class 3 “Avoids deprescribing” to 4.93 (1.16) for Class 1 “Trusts their doctor” using a 6-point Likert scale with scale anchors 1 = strongly disagree and 6 = strongly agree. The direction of this effect persisted after controlling for demographic and contextual factors (Table 6). Finally, we calculated the probability of latent class membership by level of agreement with stopping simvastatin (Figure 2). There was an increased probability of Class 1 “Trusts their doctor” membership as scores increased, indicating stronger agreement with stopping the medicines. There was a small increase in the probability of Class 2 “Makes own decisions” membership between the scores of 1 and 3 and then it was consistent from 4 to 6. There was a decreased probability of Class 3 “Avoids deprescribing” membership as scores increased, indicating strong disagreement with stopping the medicine. Finally, there was a constant probability of Class 4 “Medicines not important” membership across scores, which may reflect the lack of importance they place on medicines. Overall, the four Patient Deprescribing Typology Classes was associated with participants level of agreement with deprescribing simvastatin in the hypothetical vignette.

**Table 6.** Linear Regression Exploring Agreement With Stopping Simvastatin by Deprescribing Latent Class and demographic and Contextual Factors

Variable	Slope (95% CI)	*p*-Value
Latent class		
Class 1: “Trusts their doctor”	Ref	Ref
Class 2: “Makes own decisions”	−0.34 (−0.47, −0.20)	**<.001**
Class 3: “Avoids deprescribing”	−1.80 (−1.96, −1.64)	**<.001**
Class 4: “Medicines not important”	−0.20 (−0.37, −0.02)	**.029**
*Demographic characteristics*		
Age in years	0.01 (0.00, 0.02)	.306
Gender		
Male	Ref	Ref
Female	0.09 (−0.02, 0.20)	.106
Country		
Australia	−0.09 (−0.23, 0.06)	.251
Netherlands	−0.27 (−0.42, −0.11)	**.001**
United Kingdom	0.04 (−0.11, 0.18)	.635
United States	Ref	Ref
Education level		
Less than a Bachelor’s degree	−0.17 (−0.29, −0.06)	**.002**
Bachelor’s degree or higher	Ref	Ref
Contextual factor		
Control	Ref	Ref
Specialist prescriber	−0.45 (−0.63, −0.27)	**<.001**
Family influence	−0.11 (−0.29, 0.07)	.243
Spouse stroke	−0.55 (−0.73, −0.37)	**<.001**
Stroke image	−0.26 (−0.44, −0.08)	**.004**
Lifestyle change difficult	−0.02 (−0.20, 0.16)	.846

*Note*s. CI = confidence interval. Statistically significant at *p* < .05 and are reported in bold font.

### Discussion

Latent class analysis (LCA) was used to identify mutually exclusive deprescribing and decision-making typologies within the data. Demographic, psychosocial, and medicine-related variables were used to define and characterize the classes. This work builds on previous qualitative and quantitative research, identifying a fourth class (or typology) through LCA. We discuss the implications of these findings for refining the Patient Deprescribing Typology measure.

This LCA was part of a hypothetical vignette study conducted in four countries (AU, NL, UK, US), which focused on simvastatin (Weir et al., 2024b). Participants were asked to select combinations of the typology descriptions, which allowed us to identify patterns in the data and observe how psychosocial, clinical, and medicine-related variables influenced the differences between classes. The most common classes were Class 1 “Trusts their doctor” (41.6%) followed by Class 2 “Makes own decisions” (30.2%), Class 3 “Avoids deprescribing” (15.5%) and Class 4 “Medicines not important” (12.7%).

#### From three typologies to four classes

The influence of trust in the doctor and decision-making preferences played a key role in how the classes were formed and seemed to redistribute the patterns, leading to the creation of a fourth class. We found that the original Typology 1 “Attached to medicines” was composed of some older adults who would consider deprescribing and others who strongly preferred to continue their medicines. Likewise, the original Typology 2 “Open to Deprescribing” comprised some older adults who would consider deprescribing and others who strongly preferred deprescribing. This resulted in an additional class in the LCA. Older adults who reported high trust in their doctor and would consider deprescribing were categorized as Class 1 “Trusts doctor.” Importantly, this was the only class whose learning style was to discuss their medicines with their doctor rather than from multiple sources. Older adults who preferred to make their own decisions and would strongly prefer deprescribing were categorized as Class 2 “Makes own decisions.” Older adults who were classified as Class 3 “Avoids deprescribing” trusted their doctor but preferred to continue their medicines which was originally associated with Typology 1 “Attached to medicines.” These older adults were more likely to have real-world experience taking a statin and expressed anxiety and worry that their health would worsen in the future if simvastatin was hypothetically stopped (Figure 2).

Class 4 “Medicines not important” aligned with Typology 3 “Defers (medicine) decision-making” because they were less interested in their medicines than the other typologies. In the LCA, it is worth noting that only 12.7% of participants preferred to defer decision making to their doctor or caregiver, a finding similar to the quantitative exploration (16.6%; Stage 3). The original qualitative work (Stage 1) included older adults with variable health status, function, and frailty. Some older adults relied on care partners (who participated in the interviews) to help make medicine-related decisions. Therefore, the interviews likely captured a broader range of preferences than may be frequently expressed by older adults recruited to take an online survey. This may explain why we continue to see fewer participants being categorized as Class 4 “Medicines not important.” Future research is needed to determine whether an individual’s categorization within the Patient Deprescribing Typologies changes if their functional status declines or reliance on a care partner increases. It seems plausible that the Patient Deprescribing Typology may not be static given that older adults shift their preferences and goal orientation to maintain functioning or avoid losses (Freund, 2024; Morris et al., 2011).

A 2019 review (Kotronoulas et al., 2019) identified 13 self-report assessments focused on patients’ experiences with polypharmacy and medicine use, but only two of these considered deprescribing: the Patient Perceptions of Deprescribing (PPOD) survey (Linsky et al., 2017) and the revised Patients’ Attitudes Towards Deprescribing (rPATD) questionnaire (Reeve et al., 2016). The review noted that both assessments could be improved by greater coverage of the following content domains: “views, attitudes, beliefs, and perceptions about medicines” (rPATD questionnaire) and “patient knowledge about and understanding of medicines, and the need for information” (PPOD survey). Deprescribing attitudinal surveys could include measures of perceived medicine importance, attachment, and positive attitudes towards medicines to reflect the factors influencing patients’ medication-related decisions, as individuals may highly value their medicines, accept polypharmacy, or resist deprescribing for a variety of reasons (Kim et al., 2023). Capturing the spectrum of attitudes toward medicines and deprescribing may facilitate the development of patient-centered interventions and improve patient-clinician conversations.

The Patient Deprescribing Typology allows respondents to express their preferences for deprescribing without needing to agree or disagree, and it does not explicitly mention the doctor in relation to deprescribing preferences. The influence of the doctor should not be underestimated, as it is a key factor in patient decision-making regarding deprescribing (Vordenberg et al., 2023). Separating a question about patients’ attitudes towards deprescribing from the influence of the doctor may help reduce potential social desirability bias (Jungo et al., 2024).

#### Next steps

The authors are involved in a survey study conducted in 14 countries in Europe (Lüthold et al., 2022) which has incorporated the Patient Deprescribing Typology measure. This will shed further light on how participants differ between countries in relation to the Patient Deprescribing Typology. Also, this survey study (Lüthold et al., 2022) asked participants to reflect on their current medicine list and consider whether there were any they would like to stop. Considering deprescribing preferences for patients’ own medicine lists, as opposed to a hypothetical vignette, may result in more authentic responses from participants. Additionally, we are currently studying how older adults respond to the Patient Deprescribing Typology when they think about one specific medicine versus all their current medicines.

#### Strengths and limitations

A strength of our work is that it examined the classes in a large sample of older adults across four countries with different health care systems. In addition, this study demonstrates the utility of LCA in understanding complex relationships. Such insights would not be possible within the constraints of standard statistical approaches to data analysis, such as linear or logistic regression.

Latent class analyses are important tools in exploring underlying structures found in data. They cannot give researchers all the information they need on their own. The researchers need to estimate how many classes may be present in the data using other techniques. In this case, we used the goodness of fit statistics, the BIC (Bayesian Information Criterion), to estimate the best number of classes present. Furthermore, there is no guarantee that subjects are assigned correctly, as LCA takes a probabilistic approach. The nomenclature of the classes assigned by the researchers is somewhat arbitrary, and there is a chance that their interpretation is not completely accurate. Finally, the decisions individuals make in a vignette may not fully reflect their real-life choices, and responses could be influenced by social desirability bias. Therefore, examining the Patient Deprescribing Typology in the context of older adults considering their own medications would provide further insights.

## Conclusions

Older adults who completed an online survey about stopping medicine were classified into four distinct Patient Deprescribing Typology classes using a LCA. These findings highlight the diverse attitudes that older adults have about deprescribing decisions, emphasizing the need for tailored communication strategies in clinical settings. Additional research is needed to examine older adults’ preferences in real-world contexts to refine and enhance deprescribing interventions.

## Data Availability

The data that support the findings of this study are available upon request from the corresponding author. The data are not publicly available due to privacy or ethical restrictions. The main study was registered at Clinicaltrials.gov Identifier: NCT04676282.
